# Adenomatoid odontogenic tumor of the mandible: review of the literature and report of a rare case

**DOI:** 10.1186/1746-160X-1-3

**Published:** 2005-08-24

**Authors:** Jörg GK Handschel, Rita A Depprich, André C Zimmermann, Stefan Braunstein, Norbert R Kübler

**Affiliations:** 1 Department for Cranio- and Maxillofacial Surgery, Heinrich-Heine-University, Moorenstr. 5, D-40225 Düsseldorf, Germany; 2 Department for Pathology, Heinrich-Heine-University, Moorenstr. 5, D-40225 Düsseldorf, Germany

**Keywords:** adenomatoid odontogenic tumor, review

## Abstract

Adenomatoid odontogenic tumor (AOT) is a rare odontogenic tumor which is often misdiagnosed as odontogenic cyst. To acquire additional information about AOT, all reports regarding AOT and cited in "pubmed" since 1990 onward were reviewed. AOT accounts for about 1% until 9% of all odontogenic tumors. It is predominantly found in young and female patients, located more often in the maxilla in most cases associated with an uneruppted permanent tooth. For radiological diagnose the intraoral periapical radiograph seems to be more useful than panoramic. However, AOT frequently resemble other odontogenic lesions such as dentigerous cysts or ameloblastoma. Immunohistochemically AOT is characterized by positive reactions with certain cytokeratins. Treatment is conservative and the prognosis is excellent. For illustration a rare case of an AOT in the mandible is presented.

## 

Adenomatoid odontogenic tumor (AOT) is a relatively uncommon distinct odontogenic neoplasm that was first described by Steensland in 1905 [1]. However, a variety of terms have been used to describe this tumor. Unal et al [2] produced a list containing all nomenclatures for AOT reported in the literatures. Many different names like adenoameloblastoma, ameloblastic adenomatoid tumor, adamantinoma, epithelioma adamantinum or teratomatous odontoma have been used before to define the lesion currently called AOT. In 1999 Philipsen and Reichart [3] presented a review based on reports published until 1997 which showed some interesting aspects regarding epidemiological figures of this tumor. Since then numerous case reports of AOT have been published.

## Epidemiology

From the early 1990s onwards 65 single cases of AOT (excluding case series of more than 10 cases) have been published. The mean age was 13.2 years (range 3 until 28 years) and the female:male ratio was 2.3 : 1. The AOT was predominantly found in the upper jaw (maxilla:mandible = 2.6 : 1). Regarding the various case series published in the literature [e.g. [4-8]] and comparing these data with the single case reports mentioned above, it has to be reasoned that the AOT has a prevalence of odontogenic tumors between 1.2% in caucasian [5] and 9% in black african patients [4]. The tumor is most often diagnosed in the second decade of life and women are about twice as many affected than men. The AOT is over two times more located in the maxilla than in the mandible and the anterior jaw is much more affected than the posterior area. According to Philipsen and Reichart [3] the AOT appears in three clinico-topographic variants: follicular, extrafollicular and peripheral. The follicular and extrafollicular variants are both intrabony and account for approximately 96% of all AOTs of which 71% are of follicular type.

## Clinical features

Clinical features generally focus on complaints regarding a missing tooth. The lesion usually present as asymptomatic swelling which is slowly growing and often associated with an unerupted tooth. However, the rare peripheral variant occurs primarily in the gingival tissue of tooth-bearing areas [9]. Unerupted permanent canine are the theeth most often involved in AOTs.

## Radiographic features

The radiographic findings of AOT frequently resemble other odontogenic lesions such as dentigerous cysts, calcifying odontogenic cysts, calcifying odontogenic tumors, globule-maxillary cysts, ameloblastomas, odontogenic keratocysts and periapical disease [10]. Whereas the follicular variant shows a well-circumscribed unilocular radiolucency associated with the crown and often part of the root of an unerupted tooth, the radiolucency of the extrafollicular type is located between, above or superimposed upon the roots of erupted permanent teeth [3]. Displacement of neighbouring teeth due to tumor expansion is much more common than root resorptions. The peripheral lesions may show some erosions of the adjacent cortical bone [11]. Comparing diagnostic arruracy between intraoral periapical and panoramic radiographs Dare et al. [12] found that intraoral periapical radiographs allow perception of the radiopacities in AOT as discrete foci having a flocculent pattern within radiolucency even with minimal calcifies deposits while panoramic often do not. Those calcified deposits are seen in approximately 78% of AOT [13]. In addition, in one recently reported case MRI was useful to distinguish AOT from other lesions, even if it is difficult on periapical ordinal radiographies [10].

## Pathohistological features

Remarkably, all variants of AOT show identical histology. The histological typing of the WHO defined the AOT as a tumor of odontogenic epithelium with duct-like structures and with varying degrees of inductive change in the connective tissue. The tumor may be partly cystic, and in some cases the solid lesion may be present only as masses in the wall of a large cyst [14]. Moreover, eosinophilic, uncalcified, amorphous material can be found and is called "tumor droplets". Some tumor droplets show a homogenous matrix whereas most tumor droplets reveal electron-dense plaques [15]. Interestingly, there are a few reports about pigmented cells in AOT. However, all of these reported lesions did not show macroscopically visible pigmentation. Racial pigmentation probably plays an important role in such cases [16,17].

## Immunhistological features

During the last few years several studies have been published dealing with the immunhistological properties of AOT. Immunohistochemically, the classical AOT phenotype is characterized by a cytokeratin (CK) profile similar to follicular cyst and/or oral or gingival epithelium based on positive staining with CK5, CK17 and CK19 [18]. On the other hand the classical AOT is negative for CK4, 10, 13 and 18. Recently, Crivelini et al. [19] detected the expression of cytokeratin 14 in AOT and concluded that this probably indicate its origin in the reduced dental epithelium which is also positive for staining with cytokeratin 14 antibodies. Positive reactions for amelogenin in limited areas in AOT are also reported as well as in ameloblasts and in the immature enamel matrix [20].

Interestingly, Takahashi et al. [21] observed a positive staining for iron-binding proteins (transferring, ferritin) and proteinase inhibitor (alpha-one-antitrypsin) in various cells of AOT indicating their role to the pathogenesis of AOT. Finally, Gao et al. [22] studied the expression of bone morphogenic protein (BMP). Whereas cementifying fibromas, dentinomas and compound odontomas demonstrated a positive reaction, all AOT as well as ameloblastomas and calcifying epithelial odontogenic tumors were negative.

## Treatment and prognosis

Conservative surgical enucleation is the treatment modality of choice. For periodontal intrabony defects caused by AOT guided tissue regeneration with membrane technique is suggested after complete removal of the tumor [23]. Recurrence of AOT is exceptionally rare. Only three cases in Japanese patients are reported in which the recurrence of this tumor occurred [24]. Therefore, the prognosis is excellent.

## Case report

**Figure 1:**
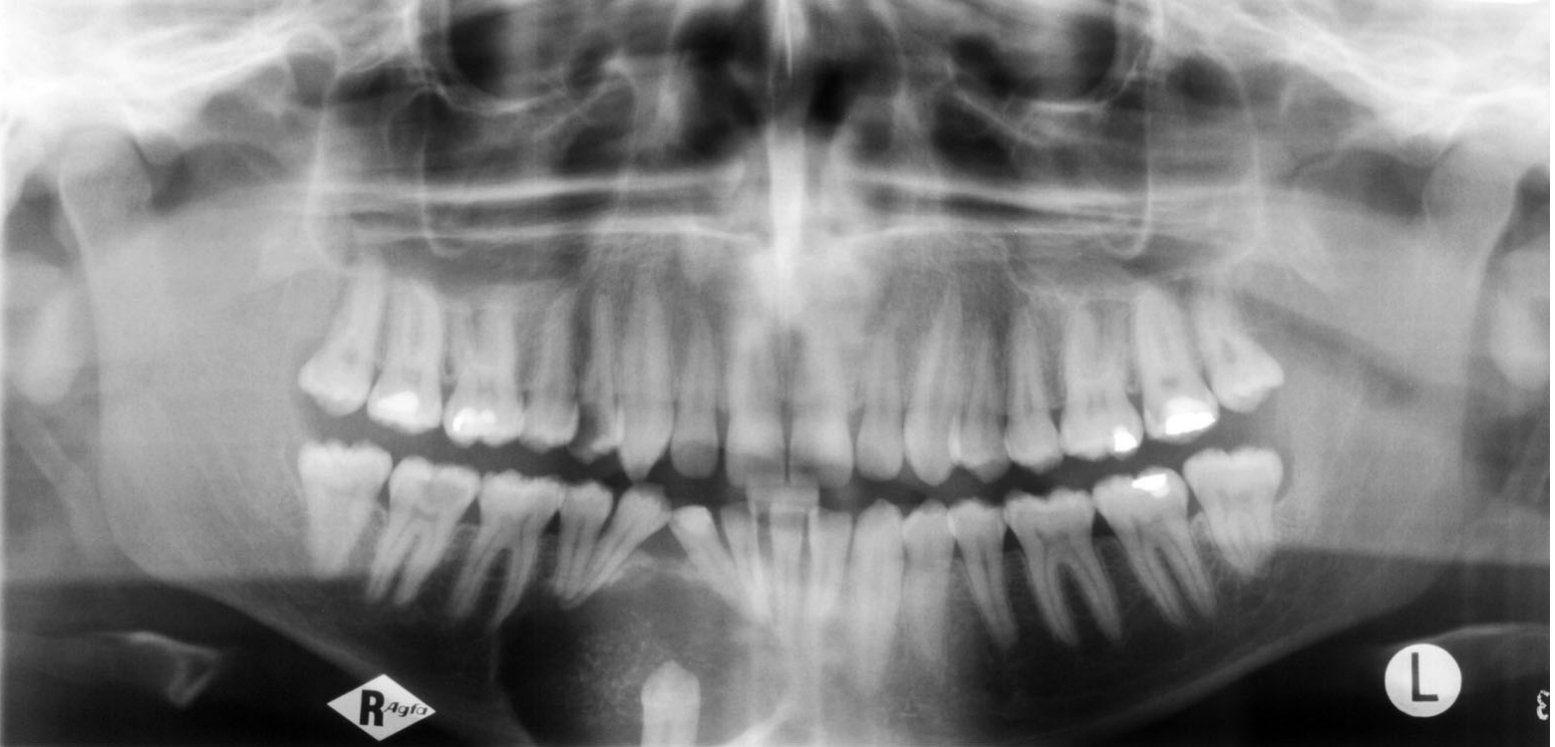
Panoramic radiograph before therapy. Unicystic radiolucent lesion in the lawer right jaw with a comparatively clear demarcation. The tooth 43 is located on the floor of this process. There are no resorption of the root apices.

A 23-year-old man was referred by his general dental practitioner. One year ago the dentist diagnosed a cyst with a ectopic lower right canine tooth by an x-ray. Beside an uneventful medical history the patient presented no conspicuous intraoral clinical findings except the absence of the tooth 43. Radiologically, he showed a 3 cm unicystic radiolucent image with a comparatively clear demarcation. The tooth 43 was located on the floor of this process. No resorption of the root apices was observed (Fig. 1).

Under general anesthesia the lesion was enucleated and afterwards filled with pelvic spongiosa. Separating the lesion from mandibular bone caused no problems. The postoperative course was uneventful.

After the operation, the specimen was fixed in 4 per cent formal saline and prepared for histological examination. Some sections were stained with haematoxylin-eosin.

**Figure 2:**
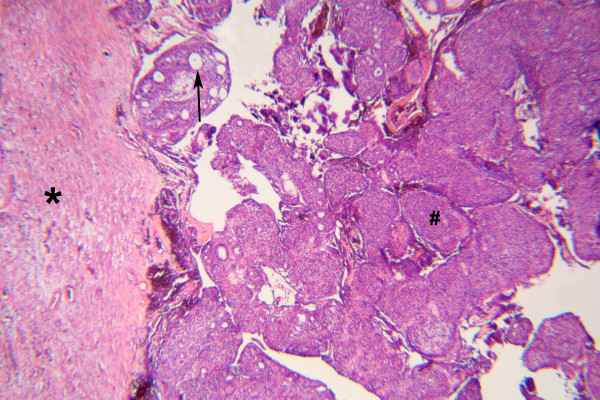
Tumor with fibrous connective tissue capsule (*). Nodular aggregates of cells (#). Duct-like structures (→). (HE × 50)

**Figure 3:**
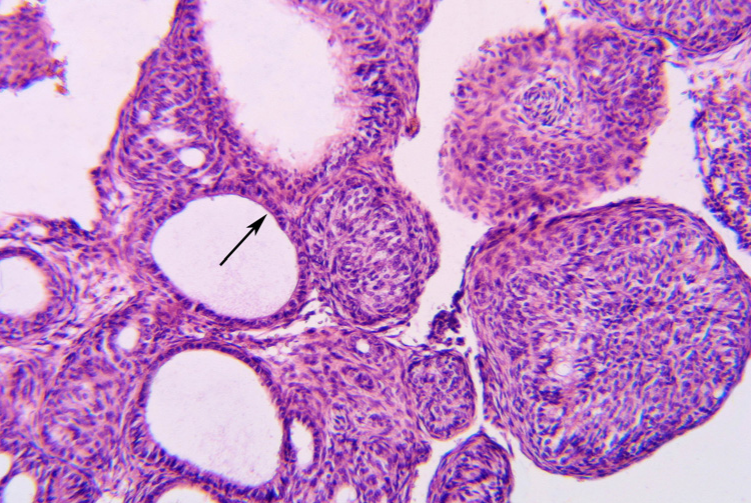
Gland-like spaces are surrounded by cuboidal to columnar cells (→). (HE × 160)

**Figure 4:**
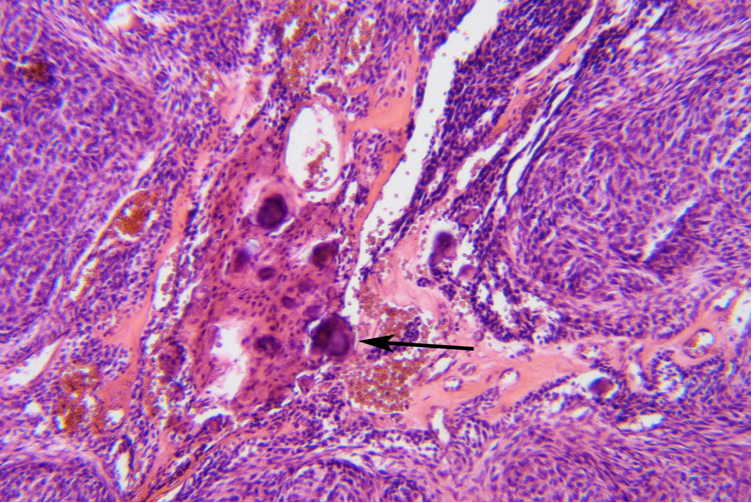
Tumor with calcified areas (→). (HE × 200)

Histologically, the tumor is solid and there is a cyst formation (Fig. 2). The epithelium is in the form of whorled masses of spindle cells as well as sheets and plexiform strands. Rings of columnar cells give rise to duct-like appearance (Fig. 3). Calcification is sometimes seen and may be extensive (Fig. 4).

**Figure 5:**
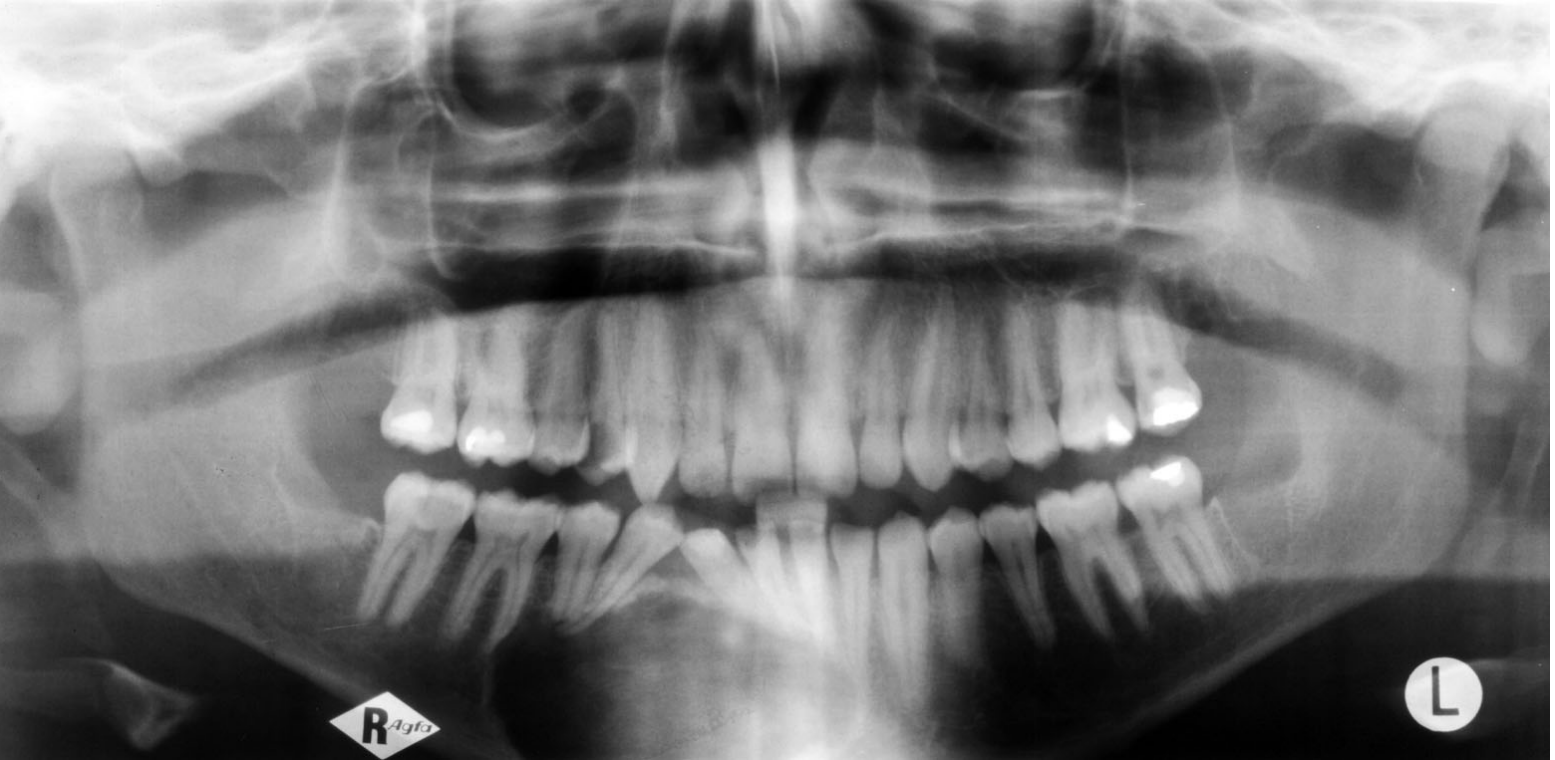
Panoramic radiograph six months after therapy. No root resorption could be observed.

Half a year after surgery a clinical and radiographic follow-up examination was performed. There was no evidence of recurrence and no apical resorption of the adjacent teeth could be observed (Fig. 5).

With respect to the age of the patient and the localization of the AOT in the lower jaw, the reported case is a rare example of this tumor entity. Beyond it our case supports the above mentioned general description of AOTs.

## Competing interests

All authors disclaim any financial or non-financial interests or commercial associations that might pose or create a conflict of interest with information presented in this manuscript.

## Authors' contributions

JH, RD and NK made substantial contribution to the conception and design of the manuscript. SB and AZ carried out the pathohistological investigations and participated in creating this part of the manuscript.

All authors were involved in revising the manuscript critically and have given final approval of the version to be published.
